# Action mechanism and cardiovascular effect of anthocyanins: a systematic review of animal and human studies

**DOI:** 10.1186/s12967-016-1076-5

**Published:** 2016-11-15

**Authors:** Jordano Ferreira Reis, Valter Vinicius Silva Monteiro, Rafaelli de Souza Gomes, Matheus Moraes do Carmo, Glauber Vilhena da Costa, Paula Cardoso Ribera, Marta Chagas Monteiro

**Affiliations:** 1 School of Pharmacy, Health Science Institute, Federal University of Pará/UFPA, Belém, PA 66075900 Brazil; 2 Pharmaceutical Science Post-Graduation Program, Health Science Institute, Federal University of Pará/UFPA, Belém, PA 66075900 Brazil

**Keywords:** Anthocyanins, Oxidative stress, Cardiovascular disease, Polyphenols, Animal models, Human study

## Abstract

Cardiovascular diseases (CVD) are an important cause of death worldwide. Anthocyanins are a subgroup of flavonoids found in berries, flowers, fruits and leaves. In epidemiological and clinical studies, these polyphenols have been associated with improved cardiovascular risk profiles as well as decreased comorbidities. Human intervention studies using berries, vegetables, parts of plants and cereals (either fresh or as juice) or purified anthocyanin-rich extracts have demonstrated significant improvements in low density lipoproteins oxidation, lipid peroxidation, total plasma antioxidant capacity, and dyslipidemia as well as reduced levels of CVD molecular biomarkers. This review discusses the use of anthocyanins in animal models and their applications in human medicine, as dietary supplements or as new potent drugs against cardiovascular disease.

## Background

Cardiovascular diseases (CVD) are the number one cause of death each year, responsible for about 17.5 million (31%) of all deaths worldwide in 2012 [1]. The most common cause of CVD-related death is coronary heart disease; the second most common cause of death is stroke [1]. Heart failure is a clinical syndrome of complex outcome, characterized by anomalies in the structure or function of the heart, impairing cardiac output [2]. The main causes of heart failure are associated with coronary heart disease, ischemia, hypertension, cardiomyopathy, atherosclerosis and type 2 diabetes [3].

In recent years, the use of polyphenols, including anthocyanins, has become common in the treatment of various diseases, including chronic and neurodegenerative conditions, as well as CVD [4]. In this regard, anthocyanins belong to flavonoid group that have several pharmacologic activity, mainly antioxidant and anti-inflammatory actions that are associated to their chemical structure [5]. Anthocyanins are a subgroup of water-soluble pigments found in the major group of flavonoids that are responsible for giving coloration that varies from the red to blue in the most varied plants, flowers, seeds, fruits and other vegetal tissues [6]. However, animals and human studies in cardiac alterations are still quite controversial and show a dual effect of the different anthocyanins and of anthocyanins-rich fruit extracts [7]. Thus, this review is focused on the current knowledge about pharmacologic actions of anthocyanins in alterations in cardiovascular system reported in human patients and animal models.

### Cardiovascular disease

The most common cause of CVD is atherosclerosis, which involves inflammation in the vessel wall, as explained above [8]. It is frequently caused by the infiltration of low density lipoproteins (LDL) the endothelium through a wall damage, once trapped in the sub endothelium, the LDL became more prone forming the oxidized LDL (oxLDL) [9, 10]. Furthermore, the oxLDL trigger the monocytes, T-cells and endothelial cells activation [11–17]. Once in the endothelium, the activation of the endothelial cells induce adhesion molecules, permeability changes, facilitating the infiltration of the macrophages and T-cells and also decrease the nitric oxide (NO) expression, leading to an increase in the vascular tonus [18, 19].

This inflammatory process involves the accumulation of several cell types, including living and dead foam cells, endothelial cells and smooth muscle cells, which contribute to the formation of atherosclerotic plaques. These process can increase the vessel thickness, decrease the lumen, leading to a more turbulent blood flow [20, 21]. In addition, foam cells in the plaque can produce proteases associated with an instability of the plaque, leading to a rupture of the plaque and creating an embolic process [22, 23]. The plaque or its embolus can compromise the blood flow in the small vessels, leading to a diminish blood, causing ischemia in the organs that this vessel is present [22]. This process is a major cause of the complications of the atherosclerosis and it is associated with principal CVD, such as coronary heart disease (CHD), stroke and peripheral arterial disease [23].

Coronary heart disease involves ischemia in the coronary artery that supplies oxygen to the heart [24]. This condition may occurs due of the buildup of atherosclerotic plaques or emboli migration in the coronary artery, which can lead to the decrease of the blood flow and the hardening of the blood vessel. This process might lead to a decrease in the oxygen influx to the heart, reducing its cardiac output or leading to cell death [25]. Stroke consists in brain damage through the ischemia by a decrease in the blood flow caused by thrombosis or embolus [26, 27]. In addition, another type of stroke is through a hemorrhagic process caused by the rupture of a brain blood vessel, that could the caused by a head injury or via rupture of the vessels due to hypertension [28].

Peripheral arterial disease involves restricted blood flow in the limbs’ peripherals vessels, especially in the legs. This condition is usually associated with the formation of atheromatous plaques in the peripheral arteries or with an embolic process in those vessels, leading to ischemia in the limbs that results in pain and committed movements [29]. The chronic nature of these diseases is associated with a remodeling response of cardiac cells and cellular environment in order to reverse the deleterious effects caused by reactive oxygen species (ROS) [30–32].

The major risk factors that can influence the development of cardiovascular diseases are: genetic predispositions, age, smoking, high cholesterol levels, high blood pressure, physical inactivity, unhealthy diet and diabetes [1]. These factors increase the likelihood of the onset of cardiovascular disease and they are related with increased oxidative stress in the body [31–35], which could increase the macromolecules damage. Accordingly, clinical and experimental studies have shown that oxidative stress is associated with the pathogenesis of cardiovascular processes, with high production of ROS in relation to antioxidant factors, which exhibit low or unchanged activity [36–39]. In addition, the heart is an organ with the lower concentrations of antioxidant in the body, which leads to increased ROS production, so it is most prone to tissue and cells damage, mainly in macromolecules, as DNA, proteins and cell lipids [36, 37, 40].

#### Biomarkers and cardiovascular disease

Cellular damage to the cardiovascular system is mainly due to lipid peroxidation caused by the action of ROS on cellular lipids, such as polyunsaturated fat acids (PUFA), phospholipids, LDL, high density lipoproteins (HDL) and very low density lipoprotein (VLDL) [20]. Thereby, these molecules can be used as biomarker; due they are associated to prognostic of CVD.

In this regard, altered PUFA levels lead to cellular dysfunction and also to the production of cytotoxic metabolites, such as unsaturated aldehydes and malondialdehyde (MDA) [41, 42]. These cytotoxic metabolites are produced by fragmentation and decomposition of lipid peroxides, especially those derived from PUFAs, not neutralized by antioxidants [43]. The most reactive unsaturated aldehyde is 4-hydroxyalkenals, a derivative of the peroxidation of an acyl group of the omega-6 (ω-6), especially the linoleic acid and the arachidonic acid. This substance has a large reactive capacity, causing damage to the mitochondrial membrane phospholipids, mainly cardiolipin [43], which is widely distributed in the inner mitochondrial membrane and plays an important role in the production of ATP [44–46], causing disorder mitochondrial homeostasis.

Others biomarkers reported are lipoproteins, LDL, VLDL and HDL, which are important predictors of CVD. In summary, studies showed that high value of LDL and VLDL associated with the high risk of CVD [47, 48], while high value of HDL are shown protective factor of CVD [49]. This imbalance can lead to a more prone environment to the oxidation of the lipoprotein in the damaged vessels increasing the concentration of oxLDL that is also associated with the high risk of CVD, especially atherosclerosis [30]. Lipoprotein (a) (LPa) is a LDL-like lipoprotein, is constituted by an apolipoprotein B (ApoB_100_) bind an apolipoprotein A (ApoA) [50, 51]. Increased levels of LPa is associated with an increased risk of CHD and atherosclerosis [52]. Its physiological function is not yet fully understood [53]. The LPa play a similar role as the oxLDL in the development of the atherosclerosis as well as to the foam cell formation [54, 55].

As inflammation is involved in induction of CVD, acute inflammatory biomarkers [e.g., C-reactive protein (CRP)] are important predictors of CVD [56, 57]. CRP is used to predict atherosclerosis risk [58] and it has shown in larger clinical trial to be a strong predictor of CVD risk [56, 58–60]. Some experimental data have shown CRP as a protective factor in the CVD, where CRP seems to bind LDL, blocking its oxidation, this process prevents the macrophages to differentiate into foam cells [61]. However, some experiments also shows that CRP activate the complement system in the atherosclerosis site, by biding to phosphocholine, so inhibiting the lectin complement pathway [57]. Despite the controversial function of CRP in the CVD, it still is a strong marker for CVD risk [57].

**Fig. 1:**
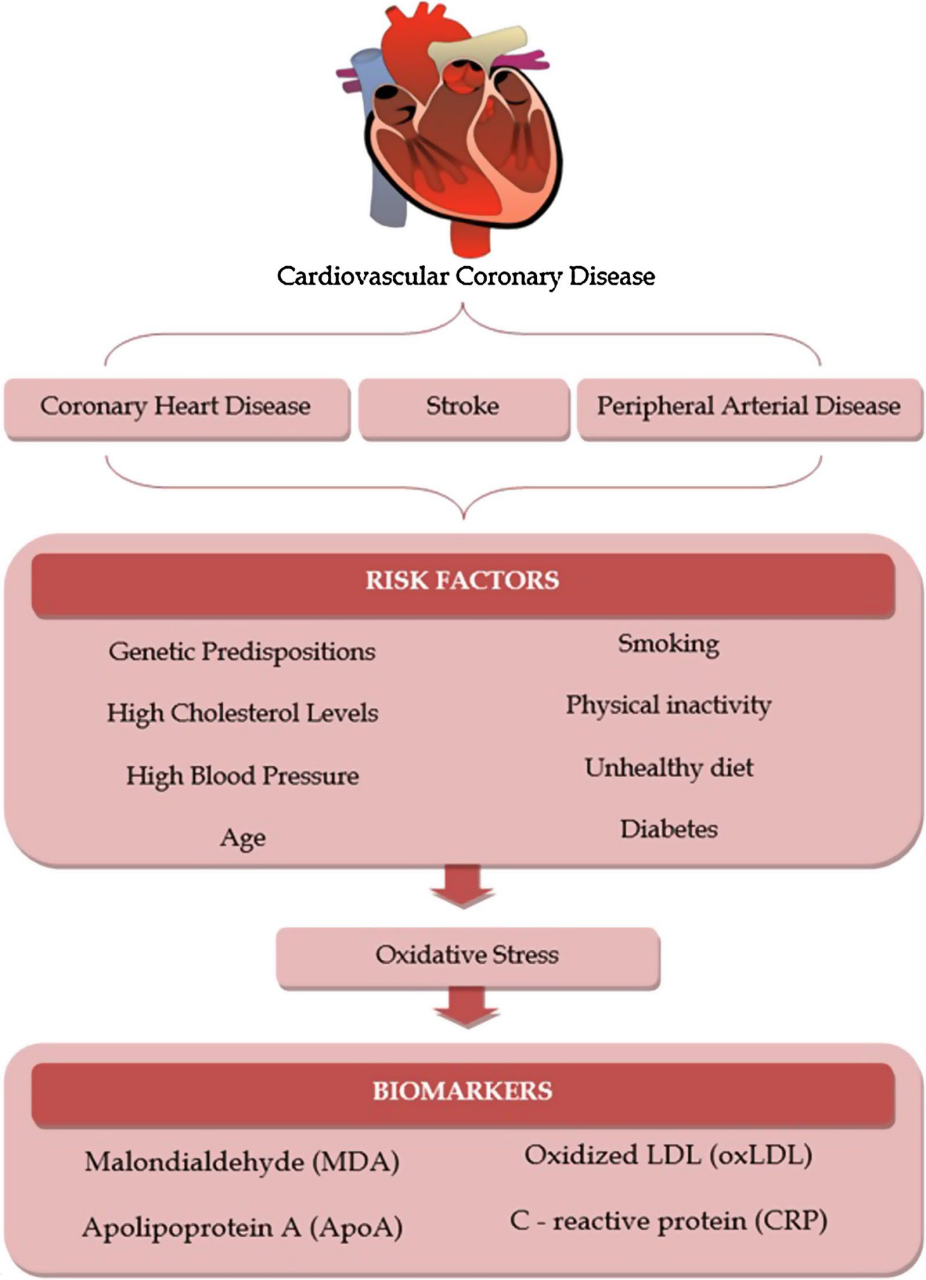
Cardiovascular diseases: risk factors and molecular biomarkers. The main risk factors of cardiovascular disease are related to the oxidative stress, generating markers that are used to predict cardiovascular risk

Cardiovascular diseases represents a major public health problem in the modern world, affecting millions of people worldwide. Its mechanism is summarized in Fig. 1. Today we have good predictor for this disease, associating its risk factor and biomarkers is possible to have an idea if a person will develop a CVD. One of the greatest problem is to prevent and to treat those diseases, being important the investigation of new therapeutic agents. The dietary polyphenols, mainly anthocyanins, have shown some cardio protector activity, as will be discussed below.

### Anthocyanins

**Fig. 2:**
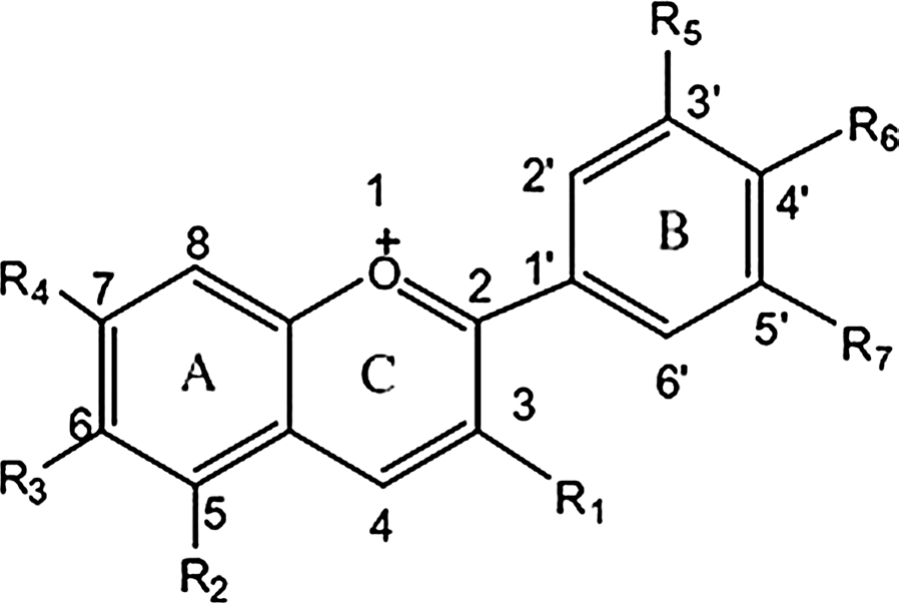
Structural body of anthocyanidins. Structural body of anthocyanidins, ion flavilium composed of an aromatic ring (*A*) condensed with a non-aromatic ring (*C*) and another aromatic ring (*B*) forming a carbon–carbon bonding

Anthocyanins are a subgroup of water-soluble pigments found in the major group of flavonoids that are responsible for colors ranging from red to blue in plants, flowers, seeds, fruits and other vegetal tissues [62], such as açaí (*Euterpe oleracea*) [63], strawberry (*Fragaria* × *ananassa*) [64], elderberry [65], chokeberry (*Aronia melanocarpa*) [66, 183–185]. Thus, almost all species of angiosperms present anthocyanins [62]. They are similar molecules that have a benzoic ring linked to a non-benzoic ring with an oxygen atom inside in a condensed way. There is yet another benzoic ring linked to those first two by a carbon–carbon bound (C–C), this structure is known as 2-phenyl benzopyrylium cation or flavylium ion [6]. In each of those rings, named A-, B- and C-rings, there are seven different radicals in all of each valences and the variation of those said radicals that will differ the anthocyanidins, as shown in Fig. 2.

Anthocyanins, the most common form of anthocyanidins in plants [67], contain the glycosylated flavylium ion [6]. There are more than 300 known anthocyanidins [68]; however, there is up to 8000 different possibilities of anthocyanins, including the different types of anthocyanidins and the glycosylated part [69].

#### Anthocyanin mechanism of action

Nowadays, the use of polyphenolics, including anthocyanins, has been important in the treatment of chronic disease, such as CVD [4]. The pharmacologic action of these polyphenolics have been associated to the chemical structure of these compounds, as shown in Fig. 1. Regarding to anthocyanins, several studies have shown their beneficial effects on CVD, inhibiting the inflammatory process, the endothelial dysfunction and NO production [70, 71]. The major proposed action mechanisms of fruits/flowers extracts or isolates anthocyanins are described below.

#### Antioxidant action of anthocyanins

The antioxidant potential of anthocyanins is regulated by differences in chemical structure, which depend on the number and position of hydroxyl groups (⋅OH), conjugation groups, the degree of glycosylation and the presence of donor electrons in the ring structure, due to the ability of the aromatic group to endure the disappearance of electrons, as shown in Fig. 1 [72, 73]. Although antioxidant activity is greatly dependent on the chemical structure of anthocyanins, some studies report that they do not possess similar activity levels for scavenging ROS/reactive nitrogen species (RNS) [73, 74].

Oxidative stress constitutes a unifying mechanism of tissue injury leading to CVD. It occurs due an imbalance between the generation of ROS and RNS and the antioxidant defense systems in the body. These reactive species attack biomolecules such as lipids, DNA, and proteins enhancing the previously established tissue damage, as well as triggering cell death pathways [75, 76]. ROS are a family of highly reactive species formed either enzymatically or no enzymatically in mammalian cells. They can cause cell damage either directly or through behaving as intermediates in diverse cell signaling pathways [76]. In the intact heart, the production of ROS occurs in three principal cells, endothelial cells, cardiomyocytes and neutrophils, or to pathway of the auto-oxidation of catecholamines [77–79]. Those free radicals are by-products of endogenous compounds or xenobiotics, provided from mechanisms like of the electron transport chain, nicotinamide adenine dinucleotide phosphate (NADPH) oxidase, xanthine oxidase (XO), metabolism of the arachidonic acid and cytochrome P-450 (CYP) [79]. Other sources of ROS may be reactions involving peroxisomal oxidases [80, 81] CYP enzymes [79, 82], NADPH oxidases [79, 82], or XO [83]. Some work also elucidate that the production of ROS can be resulted of the mechanism of the monoamines oxidases and proteins p66 [70, 84]. However, studies highlight that the major sources of ROS production in CVD are: mitochondrial, XO and NADPH oxidase pathways [84].

#### Antioxidant action of anthocyanins in ROS production

In anthocyanins, the number of free ⋅OH around the pyrone ring and the higher number of ⋅OH groups scattered throughout the molecule’s structure determine the potency of its antioxidant activity [85]. In this regard, the number of ⋅OH in the presence of C3′ and C4′ position in ring B and C3 of the C ring flavonoid basic nucleus appears to be the major structural requirement for the anthocyanins, inhibition the oxidative injury of the endothelial cells and the intracellular activity of free radicals. Moreover, the presence of methylation at positions (C3′, C4′e C3) reduce these effects. Comparing substitutions of different sugars, anthocyanins having glucose and galactose monosaccharides have higher antioxidant benefits regarding those containing disaccharides [86, 87].

The antioxidant mechanisms of the anthocyanins typically include the suppression of reactive species formation, through enzyme inhibition or the sequestration of trace elements involved in the production of free radicals [88]. It is proposed that flavonoids interrupt the chain reaction of free radicals by donating hydrogen atoms to the peroxyl radical, forming a flavonoid radical. The flavonoid radical then reacts with the free radical ending, thus, propagation chain reaction [89]. In addition, anthocyanins have an anti-peroxidative activity, as showed in some studies in that various flavonoids inhibited the lipid peroxidation of rat liver cell membrane induced both by ascorbic acid-Fe^2+^ system and arachidonic acid [90, 91].

Studies on the antioxidant effect have revealed that these anthocyanins act by different mechanisms, such as capturing free radicals and/or anions, inhibiting XO, chelating metal ions, targeting arachidonic acid and adhesion of molecules.

#### Capturing free radicals and/or anions

The phenolic structure of anthocyanins allows for the donation of a proton belonging to a free radical, which regenerates the acyl glycerol molecule and stops oxidation by free radicals. Thus, the phenol derivatives are transformed into free radicals and can stabilize and propagate without promoting oxidation reactions [92]. Along with other flavonoids, anthocyanins can directly remove the molecular species of active oxygen, including hydrogen peroxide, singlet oxygen, and superoxide, ⋅OH and peroxyl radicals [93, 94].

#### Inhibition of xanthine oxidase

Several studies have reported the inhibitory action of anthocyanins in the XO pathway. In this regard, one of the methods used to evaluate this effect in XO activity is measure the uric acid production, as described by Cos et al. [95] and Alves et al. [96] showed the inhibitory action of flavonoids on XO activity through of the increased uric acid concentration. They also demonstrated that the ⋅OH in C-5 and C-7 positions in flavonoids lead an enhanced XO inhibitory action, while the presence of these groups bonded at C-6 and C-3 considerably reduces this effect [97].

Some studies also showed that the replacement of ⋅OH with sugars in the structure of anthocyanins also could inhibit XO activity in relation to aglucone anthocyanins, indicating that steric interactions reduce the inhibitory effect on XO. Other determining factor to XO inhibitory action is the planarity of anthocyanins, which must contain the double bond between C-2 and C-3 positions and B ring that is coupled by conjugation to A and C rings. Saturation of this double bond conjugation and destroy the coplanarity of the anthocyanins are responsible for inhibitory effect on the XO enzyme. In this regard, Acquaviva et al. [98] reported the effects of cyanidin and cyanidin 3-*O*-β-d-glucoside on DNA cleavage, on their free radical scavenging capacity and on XO activity. Thus, cyanidin and cyanidin 3-*O*-β-d-glucoside showed a protective effect on DNA cleavage, a dose-dependent free radical scavenging activity and an inhibition of XO activity.

#### Chelating metal ions like iron or copper

Oxidative stress can be associated with iron storage protein, ferritin or heme proteins, which contribute to the initiation and propagation of lipid peroxidation. However, some investigators have questioned this claim, suggesting that Fe^2+^ and Fe^3+^ may be involved in iron-oxygen complex, acting as a pro-oxidant. In this context, the anthocyanins with their 3′,4′-dihydroxy groups can quickly chelate metal ions to form stable anthocyanin-metal complexes [99]. In addition, they also inhibit the oxidation of LDL induced by copper or by peroxyl radical [100–104].

Another important factor is that polyphenols with a catechol group can also bind metal ions. Bittar et al. [105] showed that anthocyanin analogues had a catechol group, which is associated to metal-binding and antioxidant properties. A study reported that the anthocyanin, eggplant peels the delphinidin-3-(*p*-coumaroylrutinoside)-5-glucoside (nasunin), has antioxidant potent activity by chelating ferrous-dependent mechanism. The findings suggest that nasunin is a potent O(2) scavenger and has protective activity against lipid peroxidation [106]. A chelating metal indirect mechanism of flavonoids was reported by Viana et al. [107] who demonstrated in vitro system that flavonoids prevent the oxidation of LDL, catalyzed by copper, the decrease in the consumption of vitamin E.

Others mechanisms of anthocyanins not yet elucidated is the inhibition of apoptosis of macrophage induced by oxidized LDL. Chang also demonstrated the ability of anthocyanins to be incorporated into the membrane and cytosol of endothelial cells, protecting them tightly against the oxidative damage induced by hydrogen peroxide and the 2,2′-azobis(2-amidinopropane) dihydrochloride [108, 109]. Anthocyanins protect the skin against ultraviolet radiation (UV) [110], helping to prevent photo-aging, combat hyperpigmentation induced by ultraviolet radiation, as well as have the ability to inhibit the synthesis of melanin by inhibiting the activity enzyme tyrosinase [111]. This strong antioxidant activity is on the one hand the result of electron deficit in its chemical structure, and, secondly, the structure conjugate of anthocyanins, allowing the electronic delocalization leads to obtaining more stable radical products requiring further studies ratio structure and activity is acting in the inhibition of this enzyme.

Regarding to CVD, in vitro and in vivo studies showed that anthocyanins inhibit the oxidative stress involved in the atherosclerotic process [112]. Since several mechanisms may be involved in this process, as the ability of anthocyanins inhibit LDL oxidation [113] and reduce oxidative injury of vascular endothelial cells [114]. The underlying mechanism remains unclear.

#### Arachidonic acid targeting: phospholipase pathways

Another pharmacologic effect of anthocyanin is its anti-inflammatory action in several disease, which is widely reported in studies about biomarkers of inflammation. In this context, lipid mediators derived from arachidonic acid, as prostaglandins produced via cyclooxygenase (COX)-2 and leukotrienes via lipoxygenases (LOX) are important targets of anthocyanins [103, 104]. Dreiseitel et al. [115] showed that cyanidin, malvidin, peonidin, petunidin and delphinidin were the most potent inhibitors of secretory-phospholipaseA2 (PLA2). PLA2 is a superfamily of esterases secreted by cells membranes that catalyze hydrolysis of sn-2 position of membrane glycerophospholipids to generate arachidonic acid as well others free fatty acids [4], precursor of prostaglandins and leukotrienes [106]. Some members of the PLA2 family is lipoprotein-associated PLA2 and secretory PLA2 [107], both the modified structures, complexed with LDL or HDL, have been shown to associate with oxLDL and activating various inflammatory pathways for atherogenesis and plaque rupture [108]. In vitro studies, anthocyanins also showed different degrees of COX-1 and COX-2 inhibition that were dependent on the number of free ⋅OH [109–113]. COX are enzymes required for the conversion of n-6 fatty acids, mostly arachidonic acid, to prostanoids that play an important role in inflammation [116]. Cyanidin has high inhibitory activity in these enzymes [109, 114].

Another important enzyme inhibited by anthocyanins is LOX. In this sense, Knaup et al. [117] tested many distinct anthocyanins and the Dp-3-*O*-glucoside and Dp-3-*O*-galactoside showed better LOX inhibition, due to combinations of radical scavenging, binding to the hydrophobic site of the LOX and/or an interaction with hydrophobic fatty acid substrate [118]. LOX uses arachidonic acid as substrate, catalyzing four different reactions such as 5S, 12R, 12S or 15S oxygenation [117]. The oxygenated substrates of these enzymes initiate biological reaction, activate cellular signaling through surface receptors or are metabolized to potent lipid mediators [119]. Kuhn et al. [120] highlighted the LOX influences in inflammatory process, atherogenesis, and hypertension.

#### NF-κB, TNF-α pathways targeting and adhesion molecules

Chronic inflammation is typical in vascular endothelial dysfunction triggered by the activation of certain factors such as nuclear factor (NF)-κB, which is functionally dependent on the cellular redox state. In this context, the tumor necrosis factor (TNF)-α activates NF-κB signaling transduction what is considered involved in the pathogenic of atherosclerosis [121]. Thereby, anthocyanins also act inhibiting NF-κB and NF-κB -dependent mediators. Paixão et al. [105] showed that malvidin-3-glucoside could suppress pro-inflammatory mediators through of the NF-κB inhibition in bovine arterial endothelial cell. Another research showed that malvidin inhibited the TNF-α, and it blocked the MCP-1, ICAM-1 and VCAM-1 expression induced by TNF-α [122]. These authors also reported that the malvidin inhibited the p65-subunit NF-κB, suggesting that malvidin could block the degradation of IkB, a cytoplasm protein that regulates NF-κB. Others anthocyanins also have been reported to inhibit the TNF-α [123–126] and NF-κB [127], as cyanidin-3-*O*-glucoside. Limtrakul et al. [128] evaluated the anti-inflammatory effect of black rice whole grain extracts (BR-WG-P), rich in anthocyanin constituents, according to their finds BR-WG-P could inhibit NF-κB and AP-1, pathways that controls pro-inflammatory mediators such as inducible NO synthase (iNOS), COX-2, interleukin (IL)-6 and TNF-α.

#### NO targeting

Vasodilation is a process to increase blood flow through endothelial cells that release vasodilators such as prostacyclin and NO, which has great influence on vascular tone [22]. It is also released by endothelium cells molecules that opposite NO function called vasoconstrictor molecules such as endothelin-1 (ET-1) and angiotensin-2 [21, 22]. The anthocyanins also have inhibitory action in vasodilators. In this regard, the cyanidin-3-glucoside (C3G) showed to be an ONOO⋅ scavenger and inhibitor of various ONOO· induced oxidative process, such as DNA strand breakage, tyrosine nitration and suppression of mitochondrial respiration [129]. Another study using extract of blackberry, which has 88% of C3G of its total anthocyanin content, data showed that this extract suppressed the NO production, suggesting that this effect was due to majority of C3G in the extract [130]. Others studies related that the C3G inhibited the nitric oxide [131, 132]. C3G, delphinidin-3-glucoside and pelargonidin-3-glucoside also were tested and they protected the ONOO· induced apoptotic on endothelial cells [133]. All anthocyanin prevented ONOO· injury on endothelial cells through disrupting mitochondrial apoptotic pathway and inhibition of the Bax nuclear translocation [133]. Martin et al. [134] showed that delphinidin and cyanidin led down-regulation of the eNOS and ET-1 expression, preventing endothelial cells apoptosis.

#### Induction of Nrf2 transcription triggering heme oxygenase-1 expression

Some studies have also reported that anthocyanins had a stimulatory effect in the nuclear factor erythroid 2-related factor (Nrf2) pathway. The Nrf2 is an inducible transcription factor with a high sensitivity to oxidative stress located in the cytoskeleton, which is widely expressed in organs with hyperoxia consumption, such as the muscle, heart, vasculature, liver, kidney, brain, lung, skin, and digestive tract [135, 136]. In humans, Nrf2 protein has 605 amino acids, a molecular weight of 66 kDa and contains a basic region leucine zipper-type (bZIP) motif in its C-terminal domain [135, 136]. Under normal conditions, Nrf2 is associated to an actin-bound protein, Keap1, but upon exposure to chemicals (often electrophiles) or ROS, the ubiquitin proteasome pathway (ubiquitin E3 ligase) promotes the Nrf2 degradation, thus the stabilized Nrf2 accumulates in the nucleus and transactivates the antioxidant response elements (ARE)-regulated target gene [135–137]. In this regard, Nrf2 is a key regulator of endogenous antioxidant and protective defense, including glutathione S-transferase (GST) and peroxidase (GPx), NAD(P)H: quinoneoxidoreductase1(NQO-1), hemeoxygenase-1(HO-1), glutamate cysteine ligase (GCL), γ-glutamylcysteine synthase (GCS) and glucose 6-phosphate dehydrogenase (G-6PDH) [135, 136, 138].

Therefore, the Keap1-Nrf2 system protects cellular proteins and DNA from oxidative damage caused by ROS and electrophiles, due an upregulation of antioxidant enzymes and decreased sensitivity to oxidative stress damage related to inflammatory reactions, respiratory system and, cardiovascular diseases [135, 136]. The physiological oxidative stress levels, related to CVD, activate weakly the Nfr2 [135]. Thus, pharmacological interventions to enhance the efficiency of the induction of Nfr2 provide homeostatic mechanisms to increase its antioxidant activity.

In this regard, Soreti et al. [139] showed that endothelial progenitor cells treated with high and low concentrations of C3G produced HO-1 in a dose-dependent manner. Therefore, these authors suggested that the endothelial protection mechanism of C3G could also be associated with HO-1 induction, antioxidant enzyme regulated by Nrf2. In addition, another study with *Aronia melanocarpa* showed high levels of HO-1 in endothelial progenitor cells cultured with angiotensin II, and also the enhanced level of Nrf2 in a concentration-dependent manner [140]. Thus, an important mechanism of anthocyanins’ antioxidant activities could be due not only by its polyphenols but also with the activation of the Nrf2 that promotes the induction of HO-1. Recently, Pantan et al. [141] reported that C3G anthocyanin may be used in combination with low doses of statin, which may be alternative treatment for atherosclerosis due both to the anti-inflammatory and antioxidant properties attenuating oxidative stress. Data showed the synergistic effect of atorvastatin and C3G enhanced the activation of the Nrf2 signaling pathway, promoting the activation heme oxygenase (HO-1).

### Studies in animal models involving anthocyanins in cardiovascular diseases

Animal models have proven successful in validating hypotheses with high accuracy when compared to human trials. The compilation of the animal models in the next sections is showed in Table 1.

**Table 1:** Effect of anthocyanins in animal models

Model	Pharmacological effect	References
Rats	↓ SBP	[142–147]
	Vasodilation	[148–152]
	↓ Total cholesterol	[153, 156]
	↓ LDL	[154, 156, 158]
	↓ VLDL	[154]
	↑ HDL	[154, 155]
	↓ Total triglycerides	[153, 156–158]
	↑ PUFA	[153]
	↓ MDA	[154]
	↓ GSH	[155]
	↓ Lipid peroxidation	[155]
Mice	↓ Total cholesterol	[147, 162–165]
	↓ LDL	[163]
	↑ HDL	[163, 164]
	↓ Total triglycerides	[147, 164, 165]
	↓ Lipid peroxidation	[163]
	↓ Atherosclerotic lesions	[161, 166]
	↓ TNF-α	[166]
	↓ NF-κB	[166]
Rabbits	↓ Total cholesterol	[170]
	↓ LDL	[170]
	↑ LDL	[169, 172]
	↓ VLDL	[169]
	↑ HDL	[170, 171]
	↓ Total triglycerides	[172]
	↓ MDA	[168]
	↓ CRP	[170]
	↓ GSH	[170, 172]
	↑ ApoE	[170]
	↓ Atherosclerotic lesions	[172]

*SBP* systolic blood pressure, *PUFA* polyunsaturated fatty acids, *LDL* low-density lipoprotein, *VLDL* very low density lipoprotein, *HDL* high-density lipoprotein, *MDA* malondialdehyde, *CRP* C-reactive protein, *GSH* glutathione, *ApoE* apolipoprotein E, *TNF-α* tumor necrosis factor-α

#### Rat model

As described above, anthocyanins have various pharmacologic actions. Here, we report several studies involving the cardiovascular system performed in rat models. Shaughnessy et al. [142] showed the inhibition of systolic blood pressure (SBP) in spontaneously hypertensive stroke-prone rats (SHRSP) after the treatment with blueberry-enriched diet (BB). The results demonstrated that after 8 weeks of treatment with BB, the SHRSP + BB group presented SBP of 178 ± 15 mmHg, while the control group (SHRSP) has SBP of 216 ± 11 mmHg. These authors concluded that food intake as BB may be used to combat hypertension and cardiovascular disease prevention. Prior studies also have demonstrated a reduction in blood pressure after diet rich in antioxidants [143–147].

The consumption of a BB diet for 7 weeks was used by Kalea et al. [148] to investigate vascular reactivity. The vasoconstriction induced by l-phenylephrine (Phe) in aorta rings was lowest in groups fed with BB, as well as antagonism of NO synthase caused a significant increase in vasoconstriction in both groups. NO dependent vasodilation via endothelium induced by acetylcholine was higher in the experimental group. The BB diet showed vasodilator and vasoconstrictor effect on the aorta and this was dependent of the NO metabolic pathway. Other findings also observed vasodilator effect of anthocyanins in the endothelium-dependent relaxation [149–152].

An anti-atherogenic effect of grape–bilberry was demonstrated by Graf et al. [153], who reported that treatment with anthocyanin-rich grape–bilberry juice, at doses of 1.551 mg of anthocyanins/L, reduced total cholesterol and triglyceride levels in the treated animal. Moreover, these authors investigated the distribution of fatty acids in plasma, and they observed decreased saturated fatty acids and increase of the long-chain n3-PUFA. Thus, the intake anthocyanins-rich juice of had beneficial effects in preventing atherosclerosis by improving endothelial function and serum lipid.

The lipid profile and oxidative stress were analyzed by Mohamed et al. [154] in animals treated with a hyper-caloric diet supplemented at different concentrations (2.5, 5 and 10%) with two species of blackberry (*Morus alba* L. and *Morus nigra*) over the course of 4 weeks. In relation to oxidative stress, the authors observed a reduction in MDA and NO levels in the *Morus alba* L. group (5 and 10%) and the *Morus nigra* group (2.5, 5 and 10%). These fruits also increased the total antioxidant capacity (TEAC) at all evaluated concentrations of blackberry fruit. Regarding to lipid profile, there was significant reduction of total cholesterol, triacylglycerols, LDL and VLDL and increase in HDL in plasma in relation to control group. The consumption of blackberries rich in natural antioxidant could prevent the risk of onset of cardiovascular diseases, reducing the lipid profile and the oxidative stress.

Sankhari et al. [155] used an atherogenic animal model to evaluate the effect of purple cabbage (*Brassica oleracea* L.) on oxidative stress in heart and in liver. During 8 weeks, the animals received an atherogenic diet and oral supplementation of the anthocyanin-rich extract of purple cabbage (100 mg/kg/day). The results of the ingestion of anthocyanins by atherogenic rats showed effective decrease of glutathione (GSH) and elevation of the HDL-c level in serum, as well as the decrease of the cardiac (creatinine kinase, creatinine kinase-MB, lactate dehydrogenase) and hepatic markers (aspartate transaminase and alanine transaminase) compared to atherogenic rats. In the oxidative stress in cardiac and hepatic tissues, the result demonstrated increase of the activities of superoxide dismutase (SOD), Catalase, GSH and ascorbic acid. The levels of lipid peroxidation had significant decrease in the studied tissues, suggesting the cardioprotector and hepatoprotector effects of the anthocyanins.

Zawistowski, Kopecand and Kitts [156] studied the onset of hypercholesterolemia in Wistar rats treated with an atherogenic diet, with the supplementation of a black rice (*Oryza sativa* L.)-derived, anthocyanin-rich extract (3% w/w) for 10 weeks. After this period, the treated groups with just the atherogenic diet presented accentuate hypercholesterolemia. The results analyzed showed that the addition of black rice extract was capable to decrease the total cholesterol, LDL-c and triglycerides levels. However, there was not a significant difference in the levels of HDL-c. It was analyzed the decrease of total cholesterol concentration in the liver of the treated group, but there was not any significant difference in the heart or aorta. Yang et al. [157] also analyzed the supplementation with anthocyanin extract from black rice (5 g/kg) during 20 weeks, showing the reduction of triglycerides and the absence of significant difference of total cholesterol, LDL-c and HDL-c in the groups. This data showed that the ingestion of black rice was promisor in the reduction of the lipid profile in rats with hypercholesterolemia.

Valcheva-Kuzmanova et al. [158] showed the anti-hyperlipidemic effect of *Aroniamelanocarpa* fruit juice (AMFJ) at doses of 5, 10 and 20 mL/kg, which was administered over the course of 30 days in rats treated with a hyperlipidemia diet. The serum levels of total cholesterol and LDL-c found lower levels in all the tested doses when compared with the control. Regarding the concentration of triglycerides, only the dose of 20 mg/kg of AMFJ able inhibit triglycerides. Yet, the experimental groups did not show significant difference in HDL-c. The results point to a possible heart protective effect of AMFJ, once it has in its composition phenolic compounds that might have antioxidant action, for example, the anthocyanins. Hypolipidemic and of oxidative stress effects were also shown in other studies [159, 160].

#### Mouse model

Mauray et al. [161] also performed a study with apo E-deficient mice. The mice were treated with a diet of anthocyanin-rich bilberry extract (0.2 g/kg) or fermented blueberry extract (0.2 g/kg) during 16 weeks. They observed reduction in atherosclerotic lesions in anthocyanin-rich bilberry extract (15%) and fermented blueberry extract groups (36%). The concentration of thiobarbituric acid-reactive substances (TBARS), the plasma antioxidant capacity, total cholesterol and triacylglycerol were not showed significant difference between the treated groups. In 2012, Mauray et al. [162] redid the treatment with apo E-deficient mice, however using only anthocyanin-rich bilberry extract and a control group, during 2 week. The results did not show significant difference in triglycerides concentration, plasma antioxidant capacity and level of HDL/LDL, corroborating with results found in 2009 research. However, total cholesterol levels were reduced due to ingestion of anthocyanin-rich bilberry extract. Both studies showed that the anthocyanin extract was not able to reverse lipid index and oxidative stress, but there may be some fermented anthocyanins bioactive compounds to prove its positive results.

In the same year, Wang et al. [163] used two treatment models to investigate the effect of C3G on endothelial dysfunction and atherogenesis in Apo E-deficient animals. In the prevention model, the animals were treated with doses of C3G (2 g/kg) and a cholesterol-rich diet during 8 weeks. This model showed reduction of total cholesterol levels and LDL-c, as well as high HDL-c level in experimental group; however, there was no significant difference in triglyceride levels. The ingestion of C3G promoted augmentation of relaxation of acetylcholine-induced aortic rings, and reduction of atherosclerotic lesion in 54%. In addition, theses authors also showed reduction of cholesterol concentration, 7-ketocholesterol (7-KC) and 7-KC-cholesterol, and increase of ATP-binding cassette transporter G1 protein (ABCG1) in aortic C3G animals. The oxidative stress parameters analyzed showed reduction of SOD and lipid hydroperoxide in the treated group. Mice fed with C3G had increased phosphorylation of eNOS SER1177, leading the increase of concentration of nitrite and nitrate, and lower cGMP in the aorta. Thus, the treatment showed recovery in endothelial dysfunction, reduced atherosclerotic lesion, more ABCG1 expression, lowering of cholesterol level and 7-KC and an increase in cGMP concentration compared to the control. However, no significant difference in triglyceride levels.

Zhang et al. [147] obtained similar results in diabetic apolipoprotein E-deficient mice after the animals consumed C3G (0.2%) for 6 weeks. The consumption of C3G improved the impairment of endothelial function, lipid profile and prevention or treatment of diabetic vascular complications. Xia et al. [164] also studied anthocyanin-rich extract from black rice and evaluated the lipid profile in animals. The animals were treated with 300 mg/kg of the extract during 20 weeks and showed reduction of cholesterol, triglycerides, HDL-c levels in serum in comparison to control group. Wu et al. [165] verified that the doses of 40 and 200 mg of the purified anthocyanins from mulberry (MACN) during 12 weeks was enough to reduce the levels of cholesterol, triglycerides and total lipids when in comparison to high-fat diet-treated group, acting as an important heart protector.

Anti-atherogenic effects and the reduction in inflammatory activity were analyzed by Wang et al. [166], who demonstrated the inhibition of TNF-α, vascular cell adhesion molecule 1 (VCAM-1), intercellular adhesion molecule 1 (ICAM-1) and nuclear factor-κB (NF-κB), demonstrating that ingesting protocatechuic acid can reduce the pro-inflammatory factors responsible for the worsening of atherosclerosis. Reduction of inflammation by anthocyanins was also reported by Seymour et al. [167].

#### Rabbit model

Yamakoshi et al. [168] evaluated supplementation with proanthocyanidin-rich extract from grape seeds (1% w/w) in cholesterol-fed rabbits. The experimental group showed a lower hydroperoxide and MDA levels compared to control group. However, did not affect the lipid profile, suggesting the antioxidant activity of the polyphenol.

Finné Nielsen et al. [169] studied the effects of black gooseberry juice (BGJ) and anthocyanin purified from black gooseberry juice (APE) extract, at doses of 58 mg/100 mL and 100.3 ± 12.8 mg/100 g, respectively. In the final stage of 16 weeks of treatment was observed that the APE-treated group presented elevated cholesterol level and LDL in serum, while the BGJ group presented a reduction in VLDL. Both groups presented elevation of SOD, beside the APE group elevated the glutathione peroxidase (GPx) level. There was no reduction in cholesterol accumulation the aorta of either group. The treatment with purified anthocyanin and black gooseberry juice presents conflicting results, suggesting further investigations to prove the results.

Kabiri et al. [170] studied regression in a rabbit model. The animals were submitted to protocol of hypercholesterolemic diet during 45 days, after they received supplementation of *A. caudatus* extract (150 mg/kg/dia), associated with normal diet, during 30 days. The *A. caudatus* extracts, considered an excellent source of anthocyanin and fibers, led to a meaningful regression of atherosclerosis area and decrease of total cholesterol, LDL, MDA and PCR levels in serum. In comparison with the control group, the treated group also had an elevation of apolipoprotein and HDL-cholesterol, showing an effectiveness antioxidant activity. The supplementation with *A. caudatus* extract decrease the risk factors to CVD, due the hypercholesterolemic diet. Abdel-Moemin [171] also had positive results in the increase of HDL-C and lower hydroperoxides and thiobarbituric reactive substances concentrations when was used the treatment with black rice (25 g for 10 weeks).

Hypercholesterolemic rabbits were also used as a model by Sozański et al. [172] to evaluate the effects of the components of cornelian cherries on lipid metabolism, augmentation of protein PPARα, lipid peroxidation and antioxidants. During 60 days, rabbits were exposed to standard diet plus 1% of cholesterol. The experimental groups were exposed to lyophilized diet of cornelian cherry compounds (100 mg/kg). After experimental diet, the results demonstrated that triglycerides level were diminished (44%) in treated animals compared to hypercholesterolemic control. Level of LDL and atherogenic index in plasma also were reduced in these treated animals, although it was not significant to cardiac risk and atherogenic coefficient in relation to control. The expression of PPARα and GSH activity were elevated, SOD and GPx levels did not show difference. Thus, they suggested that the lipid peroxidation in the liver was diminished, due to inclusion of cornelian cherry to the diet.

### Human studies involving anthocyanins in cardiovascular diseases

Several human studies have confirmed the findings found in animal models, although the literature remains controversial [169, 170]. Alvarez-Suarez et al. [64] treated health humans with fresh anthocyanin rich fruit. The participants should had cycles of dietary consumption of strawberry; they were advised to avoid strawberry and other polyphenols. After these 10 days, the patients received the strawberry for 30 days 500 g daily, and blood and urinary samples were collect. At the end of the 30 days, the patients were recommended to avoid strawberry for more 15 days. After these periods, again blood and urinary samples were collect to analyze. The results demonstrated that after strawberry-supplemented patients presented decreased levels of cholesterol, LDL and triglyceride. These parameters returned to baseline levels after 15 days of the strawberry supplementation. The study also showed lower levels of spontaneous and oxidative hemolysis. Thus, these authors concluded that the strawberry rich diet could partially protect the prevention of CVD.

Qin et al. [173] used capsules containing 17 purified anthocyanins from bilberry (*Vaccinium myrtillus*) and black currant (*Ribes nigrum*) on 120 subjects, divided in two groups, one taking two 80-mg anthocyanin capsules twice a day (320 mg/day) and the other using two placebo capsules twice a day. The patients were submitted to the treatment for 12 weeks. Blood parameters were measure before and after the period of 12 weeks with a fasting overnight. In the blood analysis was observed increase of HDL-cholesterol and cholesterol efflux in serum in anthocyanin group compared to placebo. In contrast, the LHL-cholesterol and plasma cholesteryl ester protein (CETP) were decreased in the Anthocyanin group correlated to placebo. In this study, the authors suggested that the decrease of LHL and increase of HDL is related the inhibition of the CETP. In other studies of this same group, using the same extract they also showed similar results to LDL and HDL, and they observed a significant increase of HDL-associated esterase/lactonase paraoxonase 1 (HDL-PON1), plasma cGMP and flow-mediated dilation. Thus, they concluded that the PON1 activity as associated to a better efflux of cholesterol and of cGMP, which can lead to improvement of the endothelium-dependent vasodilation through the activation of the NO-cGMP signaling pathway [174, 175].

Using the same anthocyanin-purified extract, Hassellund et al. [176] conducted an experiment similar to that designed by Qin et al. [173] but over a shorter period of time. They used 27 subjects divided in two groups, a control placebo and the treated group. The patients in the treated group received a daily intake of 640 mg of the purified extract for 4 weeks followed by a washout period of 4 weeks. Those patients were physically examined after those period of time, including oscillometric blood pressure measurements, laboratory assessments, also doing stress tests, the both cold pressor test and mental stress test. The extract treated group presented no significant difference in the blood pressure and stress reactivity levels in relation to the control group. This group also performed a similar study, using the same posology and the same extract, with 31 subjects with blood pressure of 4140/90 mmHg without use of anti-hypertensive or lipid-lowering medication. This research analyzed common CVD markers and markers for oxidative stress. They stated that the HDL-cholesterol was modest increased in the patients treated with anthocyanin.

Using an elderberry (*Sambucus nigra*) extract that contained 125 mg of anthocyanin, Curtis et al. [65] analyzed whether this extract had an effect on CVD biomarkers in post-menopausal women. This study involved 52 volunteers and they were separate in two groups, control and elderberry extract treated. They used a dose of 500 mg/day of the extract for 12 weeks. The participants had blood samples collect to verify the CVD biomarkers; they also analyzed kidney and liver function, to access safety. The treated group showed no significant changes in the cardiac biomarkers, neither in the liver and renal function. This data proved that the extract was safe however ineffective in altering the CVD biomarkers the period of the study [65].

In a study on blueberry, Basu et al. [177] used 66 obese patients with metabolic syndrome. The study included men and women. The patients were divided in two group; one group was treated with 50 g of freeze-dried blueberry dissolved and reconstituted in 480 mL of water and vanilla. In the control group, the patients were advised to consume water as control. The patients were treated for 8 weeks with an evaluation after each 4-week period. This evaluation consisted of anthropometric and blood pressure measurements, the assessment of dietary intake and fasting blood draws. The data showed a more significant reduction in blood pressure, oxLDL and serum MDA and hydroxynonenal; serum glucose concentration and lipid profiles showed no significant alteration. These results suggest that blueberries have a correlation with CVD markers and improve aspects of metabolic syndrome.

Basu et al. [178]. also investigated cranberry juice in a placebo-controlled trial. In this study, the authors observed changes in TEAC, oxLDL, MDA, inflammatory biomarkers (PCR, interleukin-6) and lipid profiles for subjects with metabolic syndrome. According to their finds, cranberry juice-treated patients had increased plasma antioxidant capacity, decreased oxiLDL and decreased MDA. However, in relation to PCR and interleukin-6, no significant alteration was observed in either group. Another study based on cranberry juice was performed by Dohadwala et al. [179]. The authors investigated the effect of cranberry juice in subjects with coronary artery disease. In this crossover study, all subjects were submitted to noninvasive methods to examine the effect of cranberry juice on various measures of vascular function. The data showed decreased aortic stiffness in comparison to the placebo group.

Another important study was the one performed by Cassidy et al. [180] in which were correlated the nutritional information about the habitual intake of flavonoids and the risks of development of CVDs from 43,880 men aged from 32 to 81 during 24 years. This research showed that habitual intakes as high as 613 mg/day of anthocyanins could be correlated to a 14% lower risk of development of nonfatal myocardial infarction in men, the same correlation was found in women before [181, 182].

**Fig. 3:**
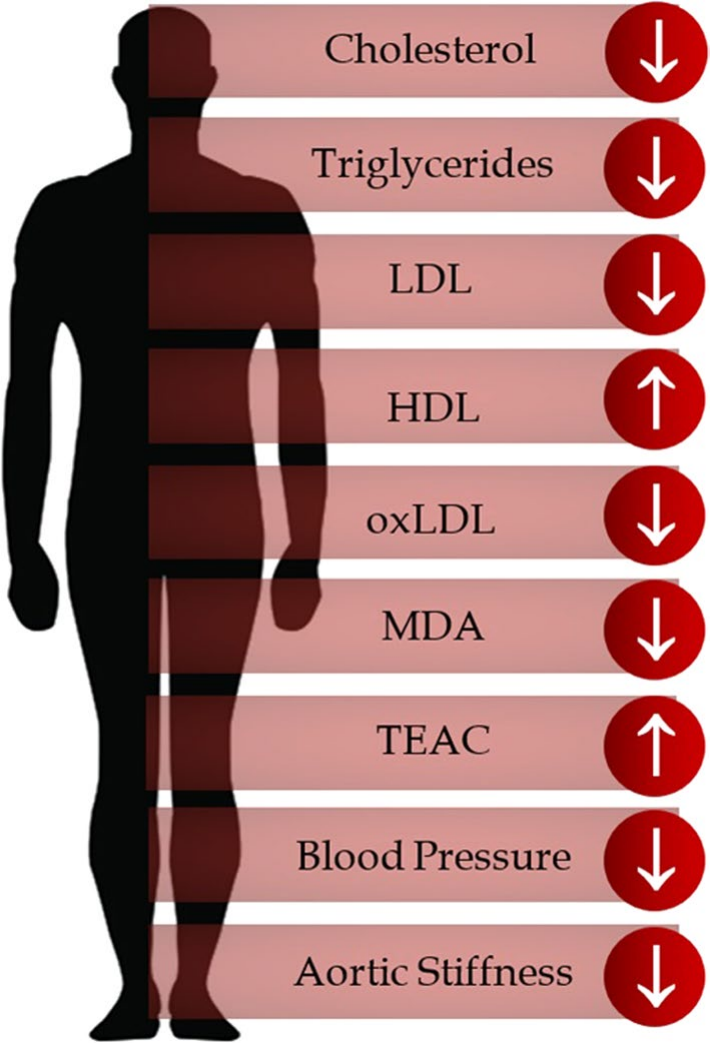
Effects and parameters altered by anthocyanins in humans

In a double-blind study Naruszewicz et al. [183] used chokeberry extract to 44 patients with mean age of 66 years (11 women and 33 men) were treated three time a day with 85 mg/day after survival of myocardial infraction, the results shown the reduction of some CVD’s markers compared with the placebo group, such as 8-isoprostanes, oxLDL, hsCRP and MCP-1. The study also stated a decrease in blood pressure and adiponectin levels. Other studies have shown that chokeberry extract have capacity of reduction of SOD and platelet aggregation levels, lower arterial blood pressure and decrease inflammation in atherosclerosis [66, 183–185]. The data collected are presented in Fig. 3.

## Conclusion

The bioactive compounds found in some plants produce positive effects that have been used in the medicinal field as potent new drugs for the treatment of several diseases, including CVD. This review showed that this fact remains true for the use of anthocyanins as potent drugs for the prevention of CVD due to inhibition the inflammatory process, the endothelial dysfunction and vasodilators production. Therefore, the major proposed action mechanisms of fruits/flowers extracts or isolates anthocyanins are antioxidant action (capturing free radicals and/or anions, inhibiting XO, chelating metal ions, targeting arachidonic acid and NF-κB, TNF-α pathways and adhesion molecules), suppression of the NO production and induction of Nrf2 transcription triggering heme oxygenase-1 expression. Human intervention studies and animal models using berries, vegetables, parts of plants and cereals (either fresh or as juice) or purified anthocyanin-rich extracts have demonstrated significant improvements in LDL oxidation, VLDL, CRP, Total Triglycerides, MDA, as well as, decreasing comorbidities. Also, improving the clinical states of patients with CVD, showing that animal studies and humans trials have been successful in demonstrating the efficacy of anthocyanins to prevent and improve the life quality of CVD patients. Despite of the potential benefit, there is still a need to standardize therapeutic strategies, such as appropriate effective dose, treatment time and relevant clinical laboratory parameters, which will allow the use of a large number of juice or purified anthocyanin-rich extracts as treatment or complement to existing treatment of CVD.

## Abbreviations

·OH: hydroxyl
Apo: apolipoprotein
CHD: coronary heart disease
COX: cyclooxygenase
CRP: c-reactive protein
CVD: cardiovascular diseases
CYP: cytochrome P-450
GSH: glutathione
HDL: high density lipoproteins
HO-1: heme oxygenase-1
IL: interleukin
iNOS: inducible NO synthase
LDL: low density lipoprotein
LOX: lipoxygenases
LPa: lipoprotein (a)
MDA: malondialdehyde
NADPH: nicotinamide adenine dinucleotide phosphate
NF: nuclear factor
NO: nitric oxide
Nrf2: erythroid 2-related factor
oxLDL: oxidized Low density lipoprotein
PLA2: phospholipaseA2
PUFA: polyunsaturated fats acids
RNS: reactive nitrogen species
ROS: reactive oxygen species
SBP: systolic blood pressure
SOD: superoxide dismutase
TNF: tumor necrosis factor
UV: ultraviolet radiation
VLDL: very low density lipoprotein
